# Quasi-stokeslet induced by thermoplasmonic Marangoni effect around a water vapor microbubble

**DOI:** 10.1038/srep45776

**Published:** 2017-03-31

**Authors:** Kyoko Namura, Kaoru Nakajima, Motofumi Suzuki

**Affiliations:** 1 Department of Micro Engineering, Kyoto University, Nishikyo-ku, Kyoto, 615-8540, Japan

## Abstract

Rapid Marangoni flows around a water vapor microbubble (WVMB) is investigated using the thermoplasmonic effect of a gold nanoisland film (GNF). By focusing a laser onto the GNF, a stable WVMB with a diameter of *~*10 μm is generated in degassed water, while an air bubble generated in non-degassed water is larger than 40 μm. Under continuous heating, the WVMB involves significantly rapid Marangoni flow. This flow is well-described by a stokeslet sat *~*10 μm above the surface of GNF, from which the maximum flow speed around the WVMB is estimated to exceed 1 m/s. This rapid flow generation is attributed to the small bubble size, over which the temperature is graded, and the superheat at the bubble surface in contact with the GNF. It is expected to be useful not only for microfluidic mixing but also for fundamental research on viscous flow induced by a single stokeslet.

Microfluidics has attracted much attention during the last few decades owing to the development of micro total analysis systems or lab-on-a-chip devices. As these devices advance, mixing techniques of small amounts of liquids have gained considerable attention^1^,^2^,^3^. A crucial point is how to apply force to the liquids packed in micro-channels, where flow is dominated by viscous effects^4^. One of the potential forces is the Marangoni force, which is induced by surface tension differential on a gas–liquid surface under temperature gradient^5^. When a portion of the surface is heated, the Marangoni force from the hotter portion to the colder portion is generated on the surface and induces fluid motion along the surface, namely Marangoni flow. Since it has been numerically proved that the stronger flow is generated using bubble surface with the smaller diameter under the same energy input^6^, Marangoni flow around a microbubble has been extensively studied, both experimentally^7^,^8^,^9^ and numerically^7^,^10^,^11^,^12^,^13^,^14^,^15^,^16^,^17^. However, precise control of the flow around microbubbles has been difficult because the Marangoni force is highly sensitive to the temperature gradient on the bubble surface. Preparation of a microbubble with exactly desired position and size is not easy, so that the position of the heater should be tuned depending on the bubble position. This makes the position-fixed heaters, such as a wire heater, unsuitable for the control of the Marangoni flow around a microbubble.

As an alternative heating method, thermoplasmonic effect of noble metal nanoparticles film has attracted recent interest^12^,^13^,^15^,^16^,^17^. The laser spot on the film can be used as a mobile and localized heat source because gold nanoparticles absorb light energy and convert it to heat energy within several picoseconds^18^,^19^,^20^. In our previous study, we demonstrated self-assembling of the gold nanoisland films (GNFs) by using a dynamic oblique deposition technique and their usefulness in thermoplasmonic control of the Marangoni flow around a microbubble^16^,^17^,^21^. Although the thickness of the GNF is thin (10–15 nm), it has a high optical absorption of 30–40% in the visible to near-infrared wavelength region because its intricate gold nanoislands distribution enhances its plasmonic absorption of the incident light (see Supplementary Fig. S1). The absorbed light is coupled to phonon within 100 ps–10 ns and rapidly heats up the material in contact with the film^19^,^20^. We created a 50-μm-thick microfluidic chamber on the film and filled it with water. By focusing a laser onto the film, water was locally heated and a microbubble with a diameter of 30–50 μm was generated on the laser spot. By tuning the laser power and the laser spot position against the bubble, a pair of counter rotating vortices were generated next to the bubble. The flow speed exceeded 10 mm/s in the vicinity of the bubble surface, and the flow pattern showed significant change depending on the laser spot position. This rapid and well-controlled Marangoni flow generation is attributed to the highly localized heat generation of GNF, which was found to be useful for particle collection and sorting^16^. Further enhancement of the flow speed is expected to be realized by reducing the bubble diameter from the discussion by Takahashi *et al*.^6^. However, in our previous experiment, the microbubble continuously grew under laser irradiation, and it was difficult to generate Marangoni flow by using a microbubble with a diameter of less than 20 μm.

The reason why the bubble diameter was not controllable is explained by the gas composition inside the bubble. Several researchers have revealed that the microbubble generated in water by the thermoplasmonic heating is an air bubble, rather than a water vapor bubble, if the dissolved gases in water are not removed by a degassing process^22^,^23^. The origin of this air bubble is the gas diffusion into the nucleated vapor bubble, which is difficult to regulate under heating. On the other hand, if the dissolved gas in water is removed by degassing process, the gas inside the nucleated bubble is maintained as pure water vapor. Therefore, bubble growth stops at the equilibrium size, where the saturation water vapor pressure and external pressure are balanced. For example, Deguchi *et al*.^22^. reported that a water vapor bubble with a diameter of even less than 10 μm can be stabilized in degassed water. If it is possible to generate a steep temperature gradient on such a tiny water vapor bubble, generation of strong Marangoni flow is expected.

In this study, we experimentally generate a water vapor microbubble with a diameter of ~10 μm in degassed water and investigate Marangoni flows induced around the bubble by using the thermoplasmonic effect of GNFs. In addition, we evaluate the force inducing the flow by using a theoretical model and show its potential as the source of a point force in microfluidics, a so-called stokeslet.

## Results

### Microbubble generation

**Figure 1:**
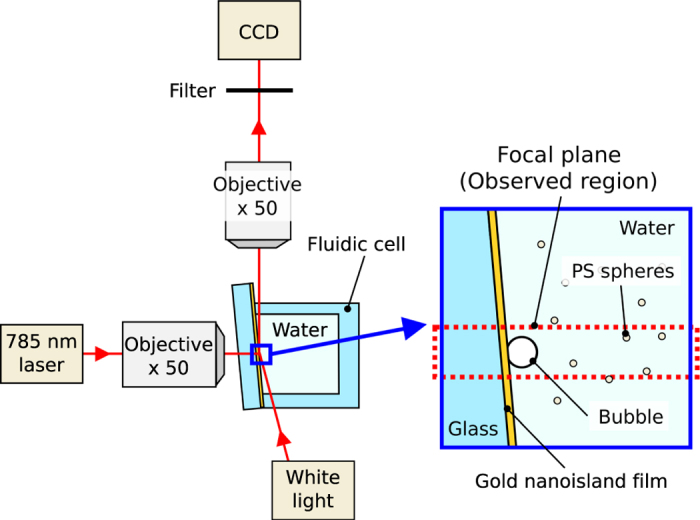
Schematic drawings of the experimental setup. The 785-nm-wavelength laser is focused on the gold nanoisland film to generate bubble and fluid motion, which is observed under white light and recorded by a CCD. PS spheres are added to visualize the flow.

Microbubble generation and fluid motion in water were observed by using the experimental setup illustrated in Fig. 1 (see the Methods section for details).

**Figure 2:**
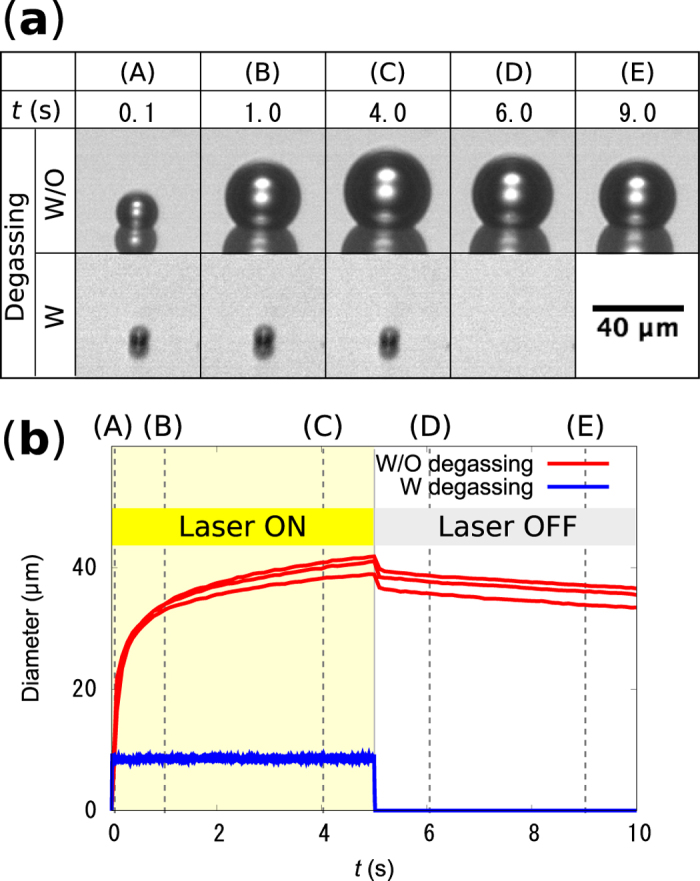
(**a**) Typical microscope images of the microbubbles created on the gold nanoisland films in water with (W) and without (W/O) degassing during (*t* = 0–5 s) and after (*t* = 5–10 s) laser heating. (**b**) The detailed time dependence of the bubble diameter, where the capital letters correspond to those in (**a**). The results of three measurements are displayed for both water W and W/O degassing.

Briefly, a GNF was fabricated on a glass substrate by using a dynamic oblique deposition technique (see Supplementary Fig. S1). Then, a fluidic cell was prepared on the GNF, which was filled with degassed water. A microbubble was created by focusing a continuous wave diode laser (wavelength: 785 nm, power: 0–31 mW) on the GNF from the rear side of the substrate. Because the gold nanoislands are relatively small compared to the laser spot, the laser spot size on the GNF determines the heated area. The laser spot on the GNF has a maximum irradiance of approximately 0.7 mW/µm^2^ and a full width at half maximum of 2–4 μm at a laser power of 30 mW (see Supplementary Fig. S2). The irradiance we used is similar to that used in the study on thermoplasmonic bubble formation with a continuous wave laser reported by Baffou *et al*.^23^. (0–1.4 mW/μm^2^). The bubble generation was observed from the direction normal to the laser incidence. Figure 2a shows successive microscope images of the generated microbubbles in water with and without degassing treatment. The laser with a power of 20 µmW is turned on at *t* = 0 s and turned off at *t* = 5 s. The detailed time dependence of the bubble diameter for *t* = 0–10 s is shown in Fig. 2b, where the capital letters correspond to those in Fig. 2a. Without degassing treatment, the bubble diameter continuously increases under laser irradiation and reaches ~40 µm within 5 s. Although the bubble starts to shrink immediately after the laser is turned off, it takes more than 20 s before the bubble disappears (see Supplementary Fig. S3). These results suggest that it is an air bubble made by water vaporization and subsequent diffusion of the dissolved air into the bubble^22^,^23^. On the other hand, in degassed water, the bubble reaches equilibrium diameter of ~9 µm within 0.02 s after the laser is turned on. In addition, the bubble disappears within 0.02 s after the laser is turned off. Because the bubble is only stable under heating, it is considered to be a water vapor bubble maintained in superheated water^22^. The thermoplasmonic effect of the gold nanoparticles allows us to maintain the local water temperature higher than 100 °C and to stabilize the tiny water vapor bubble. Because the bubble is continuously heated from the substrate side, a temperature gradient is expected to be induced on its surface. Thus, we observed the flow around the bubble by adding tracer particles to the water.

### Marangoni flow around a microbubble

**Figure 3:**
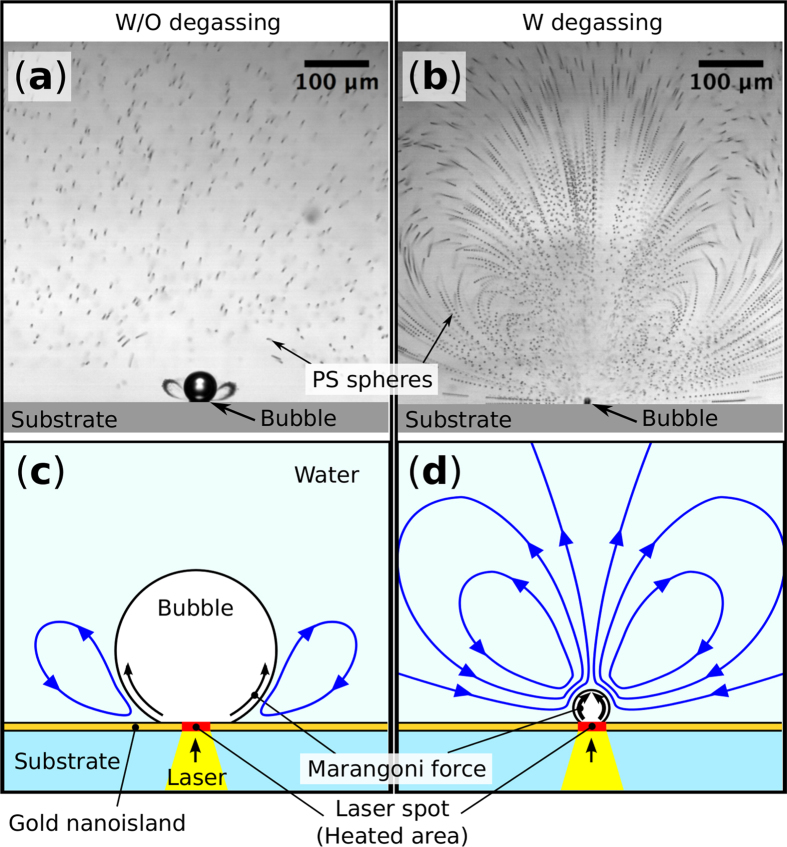
Observed flow around (**a**) the air bubble in water without degassing and (**b**) the water vapor bubble in water with degassing. A series of 20 images taken during 0.2 s are merged to trace the motion of the PS spheres in the well-developed flow. The trajectory of the PS spheres represents rapid and large rotation flow generation around the vapor bubble compared to that around the air bubble. (**c**,**d**) show rough sketches of the flow directions in (**a**,**b**), respectively. (see Supplementary Movies S1 and S2).

Figure 3a,b show typical flows observed around the heated microbubbles in water without and with degassing, respectively (see Supplementary Movies S1 and S2). The small black dots are the polystyrene (PS) spheres added to visualize the fluid motion. A series of 20 images taken during 0.2 s are merged to trace the motion of the PS spheres in the well-developed flow. Without the degassing process, rotation flows develop under the air bubble that trap the PS spheres (Fig. 3a). The observed flow is generated by Marangoni effect because the fluid is driven along the bubble surface from the hot region near the laser spot to the cold region as indicated in Fig. 3c. Similar Marangoni rotation flows with particle trapping features are reported in several papers^10^,^16^, where the authors have explained that a temperature difference of several kelvin along the bubble surface generates the flows.

**Figure 4:**
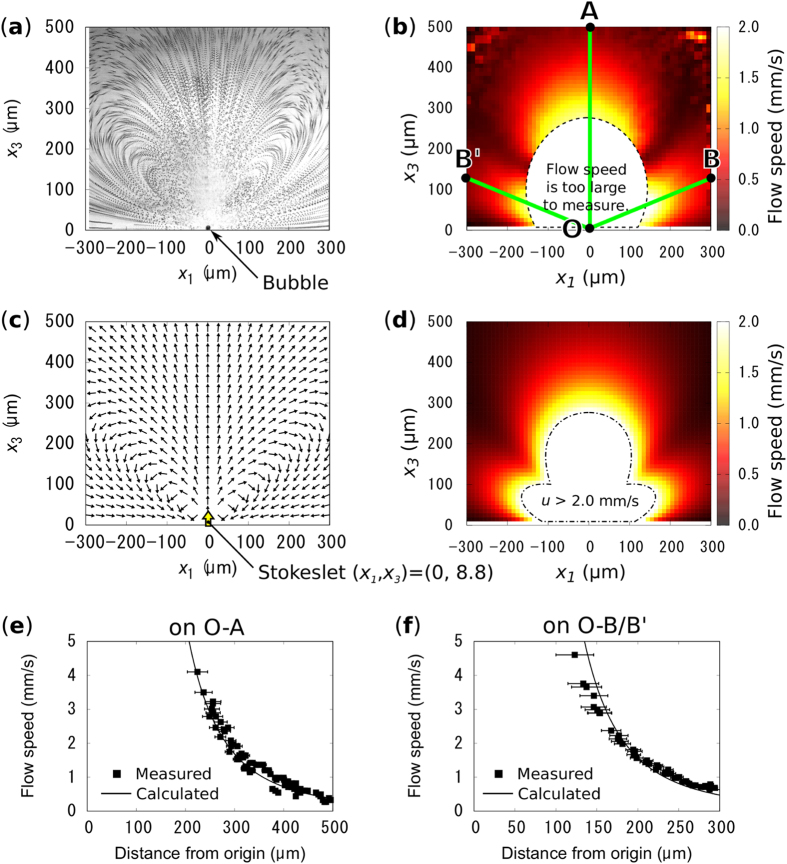
(**a**) The trajectory of the PS spheres in the Marangoni flow around the vapor bubble, which shows the flow directions distribution. (**b**) Measured flow speed distribution in the region corresponding to (**a**). (**c**,**d**) show calculated flow vector and speed distribution around a stokeslet placed above the surface of the GNF, respectively. The measurements and calculations show excellent agreement. (**e**,**f**) show manually measured flow speed (black square) on lines O-A and O-B/B’, that are indicated in (**b**), respectively. The sold black lines collespond to the calculation results shown in (**d**).

On the other hand, in degassed water, the flows observed around the water vapor bubble show significant difference from those around the air bubble. The trajectory of each PS spheres indicates strong rotation flows developed over the entire observation area (Fig. 3b). As indicated in Fig. 3d, water is drawn to the bubble and ejected in the direction normal to the substrate surface. In order to evaluate the flow vector distribution around the vapor bubble, we defined the region of interest as shown in Fig. 4a. The *x*_1_ axis is taken along the substrate surface and *x*_3_ represents the distance from the surface. The direction of the flow vector is indicated by the tangential directions of the trajectory of the spheres. On the other hand, the magnitude of the flow vector, i.e. the flow speed, was extracted from the time sequence images of the flow by using particle tracking velocimetry (LabVIEW, National Instruments). The resulting flow speed distribution is shown as a color map in Fig. 4b. Around the bubble, the flow speed is too high to measure with our CCD camera, where the displacements of the PS spheres is too large to track or images of the spheres are faint. The flow speed exceeds 1 mm/s even at 300 μm away from the bubble. This is extremely large compared to the Marangoni flow around microbubbles reported in other papers^10^,^16^.

Rayleigh–Bénard convection cannot explain the rapid flow around the water vapor bubble because the flow observation was performed in the plane normal to the direction of gravity. The phase transition of water is another possibility to generate the flow. If all of the laser power absorbed by the GNF, i.e. ~9 mW (see Supplementary Fig. S1), is used to evaporate water, the maximum amount of the evaporated water is 0.004 mm^3^/s. This value is much less than the amount of water circulated in the rotation flow, which is estimated to be more than 0.1 mm^3^/s from the measured fluid speed distribution. Therefore, the main driving force of the rapid rotation flow is not the phase transition of water. The remaining possible driving force is then the Marangoni force, which is generated by a large temperature gradient on the bubble surface. In order to evaluate the possibility to generate such a strong Marangoni flow, we estimated the force applied to water to induce the rapid flow by using a theoretical model.

## Discussion

First, we define a Cartesian coordinate system with axes *x*_1_, *x*_2_, and *x*_3_. Following Fig. 4a, a wall with no slip, i.e., the surface of the GNF, is set along the axes *x*_1_ and *x*_2_. The axis *x*_3_ is normal to the surface and the region *x*_3_ > 0 is filled with water. We assume that the observed flow around the water vapor bubble is generated by Marangoni forces applied on the bubble surface. Because the water vapor bubble is small enough compared to the generated rotation flow, we refrain from considering Marangoni force applied on a spherical surface. Instead, integration of the force over bubble surface is assumed to be applied on a point near the surface of the GNF. Direction of this point force is parallel to the *x*_3_ axis because the resultant flow is axisymmetric around the *x*_3_ axis. When the water is assumed to satisfy the Stokes flow equations, such a point force is described by one of the singularity, called “stokeslet.” We place the stokeslet, ***F*** = (0, 0, *F*), with strength of *F* [N] at ***h*** = (0, 0, *h*). The analytical solution of the flow vector distribution given by a stokeslet near a non-slip wall was reported by Blake and Chwang^24^. The flow speed, ***u*** = (*u*_1_, *u*_2_, *u*_3_) at ***x*** = (*x*_1_, *x*_2_, *x*_3_) can be written as


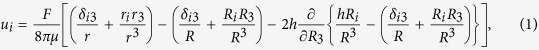


where *i* = 1, 2, and 3, *μ* is viscosity of the water, ***r*** = (*r*_1_, *r*_2_, *r*_3_) = ***x*** − ***h***, and ***R*** = (*R*_1_, *R*_2_, *R*_3_) = ***x*** + ***h***. The value of *δ*_*i*3_ is 0 for *i* = 1 or 2, and 1 for *i* = 3. In equation (1), the first term in the square bracket represents the stokeslet, the second term represents a mirror image of the stokeslet against the wall, and the third includes higher order singularities at the mirror position.

To calculate the flow speed distribution from equation (1), the values of *μ, h*, and *F* should be chosen. Because the water around the bubble should be well-stirred and superheated^23^, a constant value of 2.8 × 10^−4^ Pa · s (at 100 °C)^25^ is chosen for *μ* to avoid over estimation of *F*. The value of *h* should be in the range of 0 < *h* ≤ *d*, where *d* is the diameter of the bubble. The combination of the value of *h* and *F* is determined by substituting the measured flow speed, |***u***_***ref***_ |, at reference points, ***x***_***ref***_ into equation (1). The reference points are chosen to be (±217.5, 0, 37.5) because the flow is stable near the wall and |***u***_***ref***_ | is defined as an average flow speed of those points. Because the different combination of the value of *h* and *F* gives little difference in flow speed distribution outside of the region where the bubble exists (see Supplementary Fig. S4), we choose *h* = *d* to avoid the over estimation of *F*. The measured flow speed at the reference point is 0.7 mm/s (Fig. 4b), from which *F* is calculated to be 0.37 µN.

Figure 4c,d show the calculated flow direction and speed distribution for *F* = 0.37 µN, respectively. The flow direction distribution is consistent with the trajectory of the PS spheres in the observed flow shown in Fig. 4a. In addition, the calculated flow speed field also shows excellent agreement with the measurement (Fig. 4b), although the value of *F* is determined using the flow speed at only two reference points. A closer comparison between calculation and experimental results was performed by conducting manual flow speed measurements (see Supplementary Fig. S5). By tracing the motion of the PS spheres, we measured the flow speed distribution on the line perpendicular to the substrate surface, O-A, and on the lines 22.5 to the substrate surface, O-B and O-B’ (Fig. 4b). These lines are chosen inorder to avoid the critical region, where the flow velocity changes drastically. Figure 4e,f show the measured flow speed (black square) on lines O-A and O-B/B’, respectively. The calculated flow speed (solid black line) shows excellent agreement with the measurement, within the margin of measurement error. These results suggest that the model describes well the observed flow around the water vapor bubble. In other words, the quasi-“stokeslet”, which is one of the primitive elements of the Stokes flow, can be experimentally induced using the Marangoni flow around the water vapor bubble.

**Figure 5:**
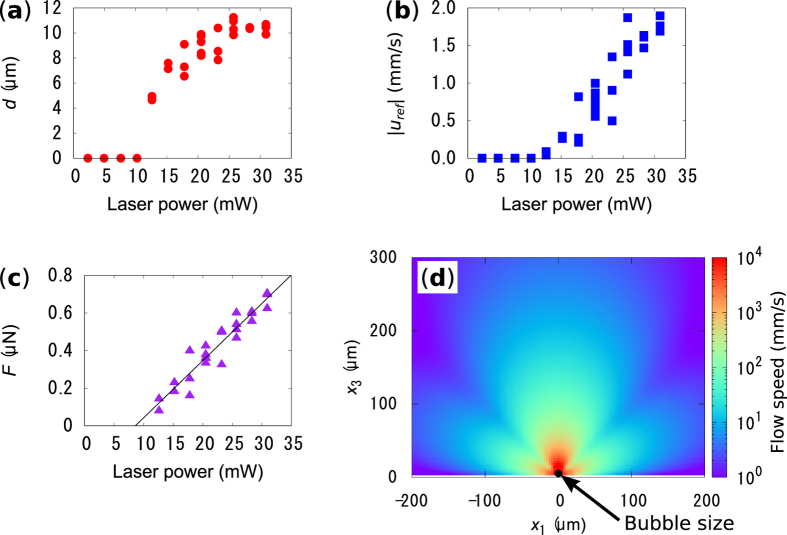
(**a**,**b**) show bubble diameters and flow speed at the reference points measured for varying laser power, respectively. (**c**) The value of *F* calculated from the experimental data shown in (**a**,**b**). (**d**) Calculated flow speed field around the bubble for *F* = 0.7 µN.

Further investigations on the flow developed around the water vapor bubble have been done experimentally and we analyzed the results by using the model described above. We observed the bubble and flow generation in degassed water under laser irradiation with various power (see Supplementary Fig. S6) and measured *d* and |***u***_***ref***_ | (Fig. 5a,b). The value of |***u***_***ref***_ | is almost zero below 10 mW, where neither bubble nor rotation flows were observed. Bubble nucleation occurs above 10 mW and the bubble diameter saturates around 11 μm. In this range of the laser power, the flow speed rapidly increases as the laser power increases, even after saturation of the bubble diameter. Therefore, the cooling effect of the generated flow on the bubble becomes larger as the laser power increases. The interplay of the cooling effect and thermal evaporation of water, due to the thermoplasmonic heating might result in the saturation of the bubble diameter, although, further study is necessary to ensure the interplay of the effects. From the sets of measured *d* and |***u***_***ref***_ | for above 10 mW, the strength of the induced quasi-stokeslet, i.e. *F*, is calculated using our model described above (Fig. 5c). The value of *F* shows linear increase as a function of laser power, for which a linear fit is shown by the black line. This result suggests that the strength of the quasi-stokeslet can be easily controlled by tuning the input laser power. The maximum value of *F* = 0.7 µN is achieved at the laser power of 31 mW, which is the upper power limit of our laser. If we assume that the Marangoni shear force is proportional to the temperature gradient and there is a uniform temperature gradient from the cold top to the hot bottom of the bubble, the temperature difference along the bubble diameter is estimated to be Δ*T* = ~300 K for *F* = 0.7 µN. Here, we used a Marangoni coefficient of ∂*σ*/∂*T* = −1.8 × 10^−4^ N/m/K^26^ and integrated the Marangoni force over the bubble surface. The estimated temperature difference is a realistic value for the following reasons. Unlike the air bubble, water around the water vapor bubble can be superheated to temperatures in excess of 100 °C. In addition, the structure of GNF changes after laser irradiation due to thermoplasmonic heating (see Supplementary Fig. S7). The Marangoni number^6^, *Ma* = ((*d*/2)^2^|∂*σ*/∂*T*||Δ*T*|)/(*αν*), calculated from the estimated temperature difference, is approximately 21, where *α* is the thermal diffusivity (1.7 × 10^−7^ m^2^/s, at 100 °C) and *ν* is the dynamic viscosity of water (2.9 × 10^−7^ m^2^/s, at 100 °C)^25^. This result indicates that heat convection is significant compared to heat conduction in our system and that the cooling effect of generated Marangoni flow plays an important roll. Further enhancement of the strength of the quasi-stokeslet is expected unless the structure of the GNF significantly changes to have low optical absorption, which is caused by the tendency of *F* to increase linearly with laser power. However, future work should focus on how such an extreme temperature gradient could be stabilized around the water vapor bubble.

Lastly, in order to show the impact of our thermoplasmonic Marangoni flow on microfluidics, we calculated the flow speed distribution near the bubble for the maximum laser power of 31 mW, where the flow speed was too high to measure experimentally. Figure 5d shows the calculated flow speed distribution developed by a stokeslet with a strength of *F* = 0.7 µN. The red portion represents the region in which the flow speed is estimated to exceed 1 m/s. This value stands out among the flows generated in micro systems^2^. This localized and extremely rapid flow generation is expected to be useful for rapid microfluid mixing, enhancement of chemical reactions, micro-engines, and so on.

## Conclusion

In summary, we investigated the rapid Marangoni flow generation around a water vapor microbubble by using the thermoplasmonic effect of GNF. Because of the highly localized heat generation of GNF and the absence of dissolved gases in water, a stable water vapor microbubble with a diameter of ~10 μm is generated by focusing a laser onto a GNF immersed in degassed water. Under continuous heating, the water vapor bubble involves significantly rapid Marangoni flows compared to the air bubble with a diameter of more than 40 μm generated in non-degassed water. The observed flow around the water vapor bubble is well-described by using a stokeslet set ~10 μm above the GNF, from which the flow speed is estimated to exceed 1 μm/s in the vicinity of the bubble. The model suggested that the superheat of the tiny bubble surface in contact with the GNF contributes to the rapid Marangoni flow generation. The thermoplasmonic Marangoni flow around a water vapor bubble is expected be useful, not only for microfluidic mixing or pumping, but also for fundamental research on viscous flow induced by a single stokeslet. Moreover, the introduction of multiple spot laser irradiation technique might allow us to generate several quasi-stokeslets at the same time and to construct complex flow in microfluidic channels.

## Methods

### Preparation of gold nanoisland films

The GNF was prepared using a glancing angle deposition technique (see Supplementary Fig. S1). Gold was deposited on a glass substrate up to an average thickness of 10 nm, during which the substrate was held at an oblique angle of 73.4° and rotated continuously and rapidly. The prepared thin film was then placed in a UV ozone cleaner (UV253S, Filgen) for 30 min in order to improve the surface wettability.

The optical absorption of the GNF at the wavelength of 785 nm is determined to be 0.43 from optical reflectance and transmittance measurements conducted using a single-beam spectrophotometer and an integration sphere (ISP-REF, Ocean Optics) (see Supplementary Fig. S1).

### Preparation of degassed water

We prepared degassed water, in which PS spheres were dispersed to visualize the flow. The water suspension of the PS spheres with a diameter of 2 μm (R0200, Thermo Scientific) was diluted with ultrapure water (18.2 MΩ cm from Millipore-Direct Q UV3, Merck) to a ratio of 1:200 (particle number density: ~1 × 10^7^ cm^−3^). Then, the diluted suspension was sonicated under water aspirator vacuum (~3 kPa at 25 °C) for 20 min. The dissolved oxygen concentration in the degassed water is 0.9 ± 0.1 mg/L, whereas in the non-degassed water, it is 3–4 mg/L (measured by DO-5509, FUSO). The degassed water was carefully transferred into a 10-mm fluidic cell cube created on the GNF and sealed with a glass cover. Following this, microfluidic observation was performed within 20 min after the cell preparation, in order to avoid the effect of gases in air, which diffuses back to the water.

### Observation of bubble formation and Marangoni flow

The prepared fluidic cell was placed to an upright microscope (M-scope, Synos) equipped with an objective lens (10×, NA = 0.26) (Fig. 1). The observed region is on the focal plane of the microscope, which is normal to the surface of the GNF and has a thickness of ~20 μm. Practically, the surface of the GNF was tilted by 5–7° from the optical axis of the microscope in order to confirm the laser spot on the film. A CW laser with a wavelength of 785 nm for thermoplasmonic heating was focused onto the GNF from the rear side of the glass substrate. The diameter of the laser spot was less than 10 μm, and the laser power at the sample surface was 2–31 mW. The fluid motion visualized by the motion of PS spheres was recorded by a CCD (HXC20, Baumer), whose exposure time and frame rate were set at 0.5 ms and 100 fps, respectively. A short-pass filter was placed in front of the CCD to eliminate the 785-nm laser source.

## Additional Information

**How to cite this article**: Namura, K. *et al*. Quasi-stokeslet induced by thermoplasmonic Marangoni effect around a water vapor microbubble. *Sci. Rep.* **7**, 45776; doi: 10.1038/srep45776 (2017).

**Publisher's note:** Springer Nature remains neutral with regard to jurisdictional claims in published maps and institutional affiliations.

## Supplementary Material

Supplementary Information

Supplementary Movie S1

Supplementary Movie S2
