# Supplementary material for: Quasi-stokeslet induced by thermoplasmonic Marangoni effect around a water vapor microbubble
Source: Sci Rep. 2017 Mar 31;7:45776. doi: 10.1038/srep45776 (PMC5374461; doi:10.1038/srep45776)
Supplement: Supplementary Information [file srep45776-s1.pdf]

Quasi-stokeslet induced by thermoplasmonic Marangoni effect around a water vapor microbubble

Kyoko Namura,* Kaoru Nakajima, and Motofumi Suzuki

Department of Micro Engineering, Kyoto University-Katsura, Kyoto 615-8540, Japan

E-mail: namura@me.kyoto-u.ac.jp

Phone: +81 75 383 3697. Fax: +81 75 383 3697

Supplementary information

1. Movies

Movie S1 and Movie S2 show the thermoplasmonic Marangoni flow around the air bubble in water without degassing and around the water vapor bubble in water with degassing, respectively. The observed region is approximately 600 µm × 650 µm and nearly normal to the surface of the gold nanoisland film. The small black dots are the polystyrene spheres with a diameter of 2 µm added to visualize the fluid motion.

*To whom correspondence should be addressed

2. Figures

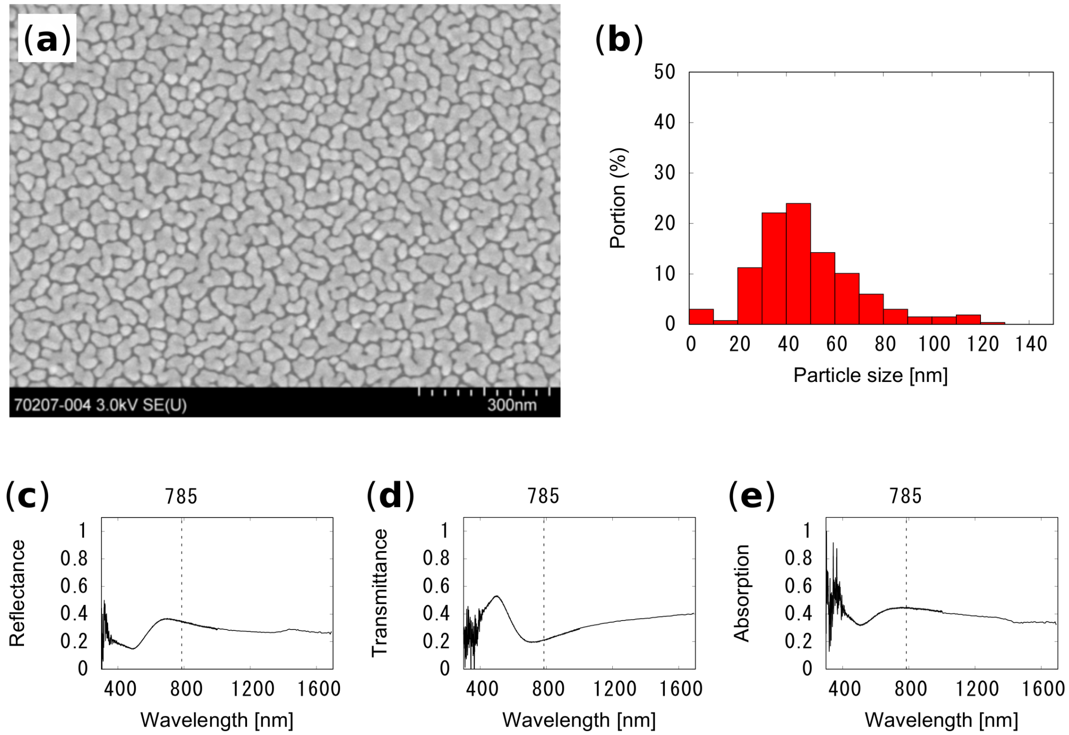

Figure S1: (a) Typical SEM image of the surface morphology of a gold nanoisland film prepared on a glass substrate by using a dynamic oblique deposition technique. (b) The size distribution of the gold nanoislands on the film. The area of each nanoisland measured from the SEM image of the film surface is associated with an equivalent round shape, of which the diameter is used to represent the size of the nanoisland. Optical (c) reflectance, (d) transmittance, and (e) absorption spectra of the gold nanoisland film. The reflectance and transmittance spectra were acquired using a single-beam spectrophotometer with a wavelength range of 300 nm $< \lambda <$ 1700 nm. The optical reflectance measurement was carried out on the sample at an incidence angle of 8° by using an integration sphere (ISP-REF, Ocean Optics). The absorption spectrum was calculated by subtracting reflectance and transmittance from unity, because the absorption of the glass substrate is negligible in the wavelength range of optical measurements. The optical absorption of the gold nanoisland film at 785 nm is 0.43.

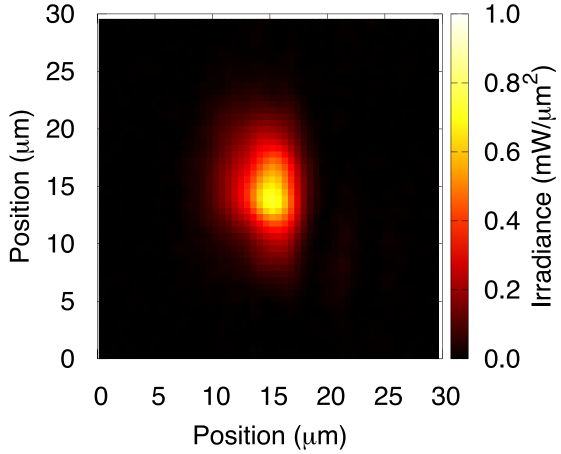

Figure S2: Irradiance distribution of the laser spot on the gold nanoisland film at a laser power of 30 mW. The laser spot has a maximum irradiance of approximately 0.7 mW/μm^2 and a full width at half maximum of 2–4 μm.

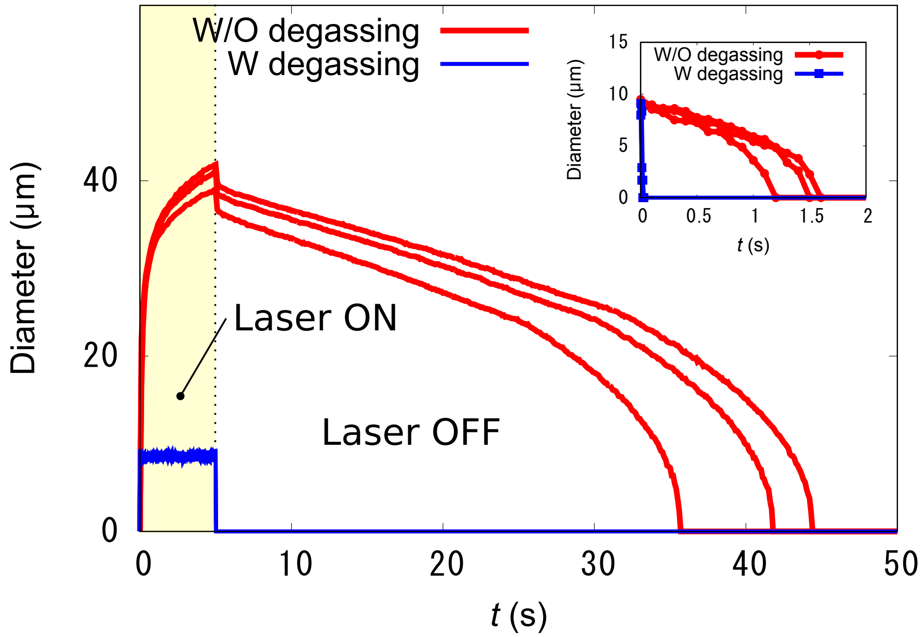

Figure S3: Time dependence of the bubble diameter, where the laser with a power of 20 mW is turned on at $t = 0$ s and turned off at $t = 5$ s. The results of three measurements are displayed for water both with (W) and without (W/O) degassing. The bubbles generated in degassed water disappear soon after the laser turned off, while those generated in non-degassed water take more than 20 s to disappear. Inset shows the lifetime of the bubbles with diameters of ~ 9 µm in water W and W/O degassing without laser irradiation. The bubbles in non-degassed water show significantly longer lifetimes than those in degassed water, although the initial bubble sizes are the same.

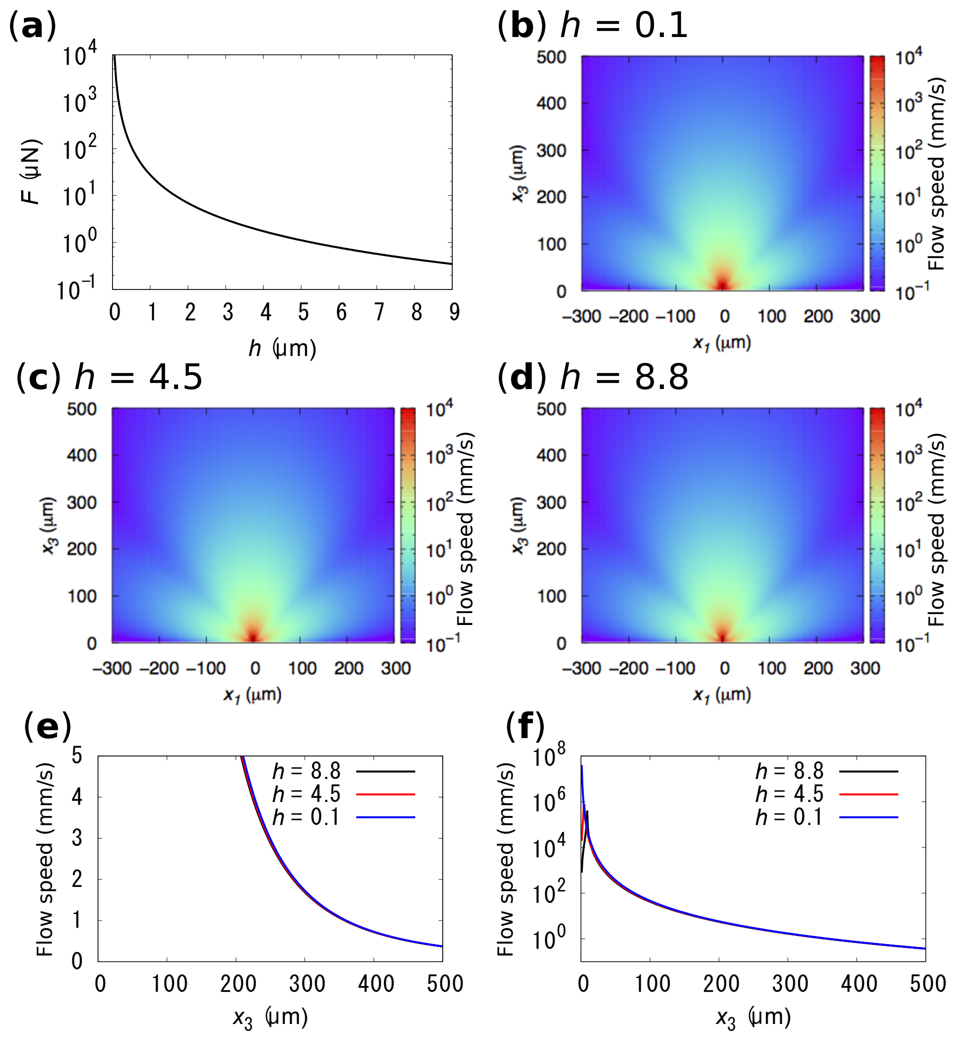

Figure S4: (a) Combination of h and F determined by substituting measured flow speed, $|\boldsymbol{u_{ref}}|$ = 0.7 mm/s, at reference points, $\boldsymbol{x_{ref}}$ = (± 217.5, 0, 37.5), into Equation 1. The smaller value of h gives the larger value of F. (b), (c), and (d) shows flow speed distributions for (h, F) = (0.1, 2800), (h, F) = (4.5, 1.4), and (h, F) = (8.8, 0.37), respectively. (e) and (f) compare those calculation results on x_3 axis. The different value of h gives little difference in the flow speed distribution outside of the region where the bubble exists.

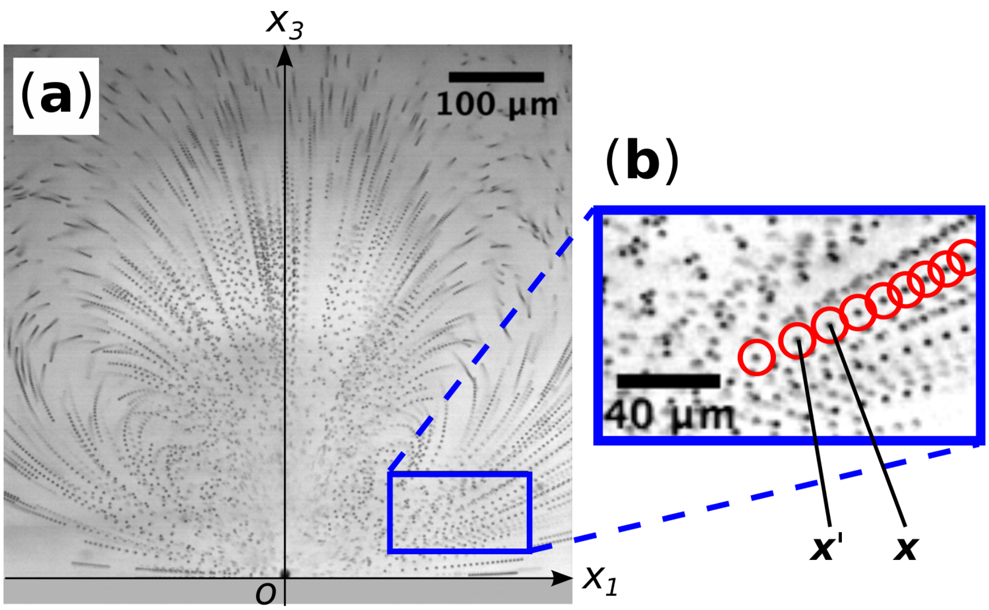

Figure S5: Typical images used for manual measurements of the flow speed. First, (a) several microscope images, taken every $\Delta t = 10$ ms, are merged. (b) The motion of the PS spheres can be traced from the string of the sphere images indicated by red circles. The flow velocity in the region between position $\mathbf{x}$ and $\mathbf{x}'$ is obtained by calculating $\mathbf{u}_{\mathbf{x}'-\mathbf{x}} = (\mathbf{x}' - \mathbf{x})/\Delta t$.

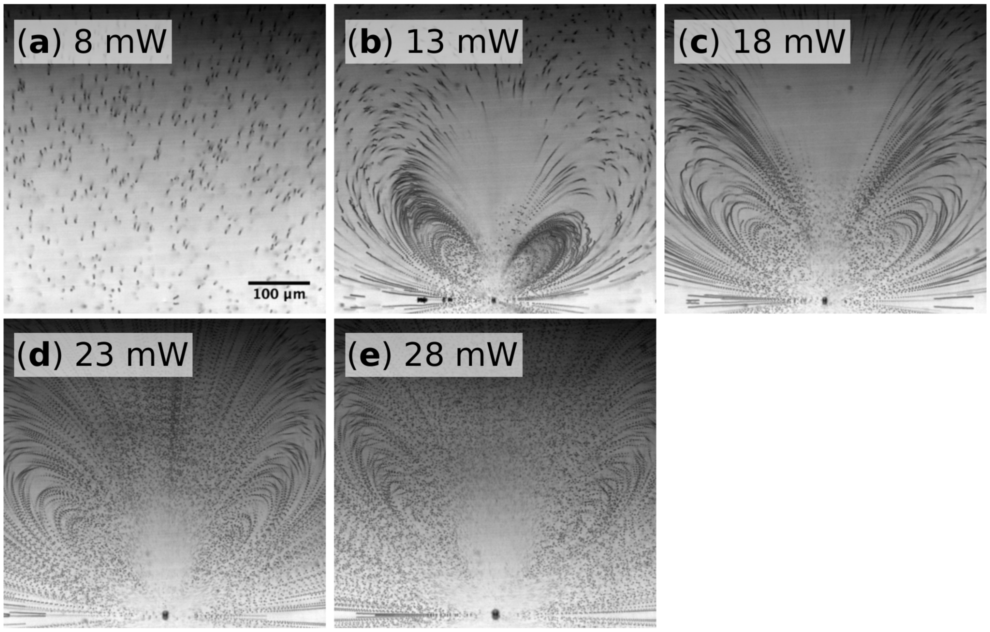

Figure S6: Observed flow in degassed water under laser irradiation with powers of 8–28 mW. A series of 100 images taken during 1 s are merged to trace the motion of the PS spheres in the well-developed flow. At the laser power of 8 mW, the generation of the bubble and the rotation flows is not observed. On the other hand, at the laser powers of 13–28 mW, the water vapor bubbles are generated and the characteristic rotation flows are observed around them.

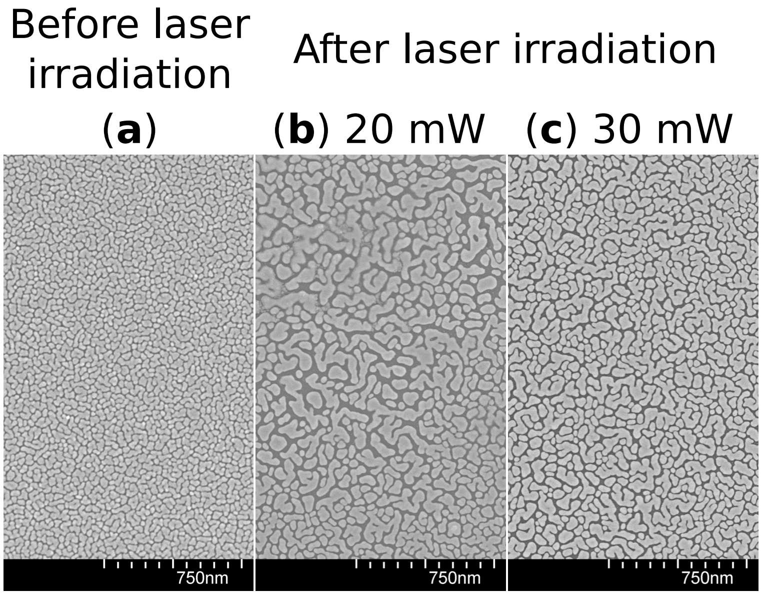

Figure S7: SEM images of the surface morphology of the gold nanoisland film (a) before and (b,c) after laser irradiation. The laser irradiation was performed in degassed water at the laser power of 20 mW and 30 mW for 5 s, during which water vapor microbubbles were formed on the gold nanoisland film. The structure of the gold nanoisland film is changed by laser irradiation, which is often observed when the film is annealed at 200–300°C.
